# Pharmacist-Led Diabetes Control Intervention and Health Outcomes in Hispanic Patients With Diabetes

**DOI:** 10.1001/jamanetworkopen.2023.35409

**Published:** 2023-09-28

**Authors:** Kimberly Danae Cauley Narain, Gerardo Moreno, Douglas S. Bell, Lillian Chen, Chi-Hong Tseng, Robert W. Follett, Samuel Skootsky, Carol M. Mangione

**Affiliations:** 1 Division of General Internal Medicine and Health Services Research, Department of Medicine, University of California, Los Angeles; 2 Center for Health Advancement, Fielding School of Public Health, University of California, Los Angeles; 3 Department of Family Medicine, David Geffen School of Medicine, University of California, Los Angeles; 4 Clinical and Translational Science Institute, University of California, Los Angeles; 5 Department of Medicine Statistics Core, David Geffen School of Medicine, University of California, Los Angeles; 6 Population Health, University of California Health, Oakland; 7 Health Policy and Management, Fielding School of Public Health, University of California, Los Angeles

## Abstract

**Importance:**

Among patients with type 2 diabetes (T2D), Hispanic individuals are more likely than non-Hispanic White individuals to develop diabetes-related complications.

**Objective:**

To examine the association of a pharmacist-led intervention (UCMyRx) with hemoglobin A_1c_ (HbA_1c_) and systolic blood pressure (SBP) among Hispanic patients with T2D.

**Design, Setting, and Participants:**

This quality improvement study used electronic health record data and a difference-in-differences study design to evaluate the association of UCMyRx exposure with changes in HbA_1c_ concentration and SBP among Hispanic patients with T2D, relative to usual care, at University of California, Los Angeles primary care clinics between February and April of 2023. The study population included patients with an *International Classification of Diseases, Ninth Revision*/*International Statistical Classification of Diseases and Related Health Problems, Tenth Revision* diagnosis of T2D, self-reporting Hispanic ethnicity, age 18 years or older, with 1 or more visits with a UCMyRx pharmacist (treatment) or 2 or more visits, 2 or more years apart, during the study window (comparison). Additionally, patients had to have the following observations during the study window (March 2, 2013-December 31, 2018): (1) a HbA_1c_ 8% or higher, anywhere between 365 days before and 14 days after the index date (date of the first UCMyRx visit or a randomly generated index date) and a follow-up HbA_1c_ measure within 120 to 365 days after the index date (n = 396) and/or (2) a SBP 140 mm Hg or higher between 365 days before and 14 days after the index date, and a follow-up SBP measure within 120 to 450 days after the index date (n = 795).

**Exposure:**

Pharmacists review laboratory results/vital signs, perform medication reconciliation, and develop personally tailored interventions to address adherence barriers and increase guideline-concordant care.

**Main Outcomes and Measures:**

Pre– to post–index date changes in HbA_1c_ and SBP.

**Results:**

Of the 931 unique patients with T2D analyzed, the mean (SD) age was 64 (14.1) years, and 552 (59.3%) were female. In adjusted analyses, having 1 or more UCMyRx visits was associated with a reduction in HbA_1c_ concentration (β = −0.46%; 95% CI, −0.84% to −0.07%) but no change in SBP (β = −1.71 mm Hg; 95% CI, −4.00 to 0.58 mm Hg).

**Conclusions and Relevance:**

In this quality improvement study of UCMyRx among Hispanic patients with T2D, a negative association was observed between UCMyRx exposure and HbA_1c_ concentration but not SBP. Pharmacist-led intervention may be a strategy for improving outcomes among Hispanic patients with T2D.

## Key Points

**Question:**

Is a pharmacist-led intervention focused on improving medication adherence and guideline-concordant care associated with improvements in hemoglobin A_1c_ concentration and systolic blood pressure among Hispanic patients with type 2 diabetes, relative to usual care?

**Findings:**

In this quality improvement study of 931 Hispanic adults, exposure to the intervention was associated with a statistically significant reduction in hemoglobin A_1c_ concentration but no change in systolic blood pressure.

**Meaning:**

These findings suggest that pharmacist-led intervention may be a strategy for improving some outcomes among Hispanic patients with type 2 diabetes.

## Teaser

This quality improvement study examines the association of a pharmacist-led intervention with HbA_1c_ and systolic blood pressure among Hispanic patients with diabetes.

## Introduction

Relative to non-Hispanic White (hereafter, White) individuals, Hispanic individuals with type 2 diabetes (T2D) are more likely to develop end-stage kidney disease and retinopathy and require lower-extremity amputations.^1^ Among the factors contributing to disparities among Hispanic patients with T2D is lower levels of treatment intensification, relative to White patients.^2^ Another contributor to worse T2D outcomes among Hispanic individuals, relative to White individuals, is reduced adherence to diabetes medications.^3^

Pharmacist-led intervention, a delivery system design approach that uses clinical pharmacists to address care quality and patient self-management behavior, may be an effective strategy for improving cardiovascular disease risk factors among Hispanic patients with T2D. Studies of pharmacist-led interventions among Hispanic patients in community settings and federally qualified health centers have also demonstrated the benefit of these types of interventions.^4,5,6,7^ However, these studies have been largely descriptive.

In January 2012, University of California, Los Angeles (UCLA) began the UCMyRx initiative, which now exists in 38 primary care clinics. The UCMyRx initiative involves embedding clinical pharmacists trained in motivational interviewing into primary care practices to comanage patients with complex care needs along with their primary care physicians (PCPs). Individuals can access the UCMyRx program in several ways, including by PCP, clinical care coordinator, or self-referral. Additionally, individuals in the UCLA Diabetes Registry, meeting 1 or more of the following criteria: (1) a hemoglobin A_1c_ (HbA_1c_) concentration of 9% or higher, (2) a systolic blood pressure (SBP) of 140 mm Hg or higher, (3) a low-density lipoprotein level of 130 mg/dL or greater, and (4) receiving 5 or more prescription medications, are contacted to schedule a visit with a UCMyRx pharmacist. In the initial UCMyRx visit, clinical pharmacists review vital signs and laboratory results, order laboratory tests as needed, perform medication reconciliation, assess medication adherence using a standardized survey, and, based on the results of the survey, implement a personally tailored intervention to improve medication adherence (Table 1). For example, survey responses that indicate out-of-pocket costs as a barrier to adherence will prompt the pharmacist to look for less expensive therapeutic options, patient-assistance programs, and generic substitutions. Dietary and physical activity counseling are also provided if indicated.^8,9^ Cardiovascular disease risk factor management is based on the American Diabetes Association, Joint National Committee, and Adult Treatment Panel guidelines and has been updated as new iterations of the guidelines have become available.^10,11,12^ To facilitate in-person visits for non-English-speaking patients, a mobile tablet is used for in-person/video interpreter services, and telephone interpreters are used for any follow-up via telephone. The results of all assessments and recommendations are communicated to the PCP through the electronic health record (EHR).^13^ Once the PCP reviews the note and documents agreement with the recommendations in the EHR, the pharmacist is able to directly prescribe or discontinue medications and order laboratory tests as needed. The pharmacist schedules follow-up visits with the patient and supplements the visits with virtual visits, emails, and phone calls as needed. Pharmacists are instructed to use their clinical judgment in terms of how often to bring patients in for visits. The objective of this study was to evaluate the association between UCMyRx exposure, HbA_1c_ concentration, and SBP among Hispanic primary care patients with T2D.

**Table 1.** Promoting Adherence Through Tailored Interventions

Barrier	Intervention
Out-of-pocket costs	Therapeutic substitutions, drug assistance programs, $4 generics, mail order prescriptions
Refill issues (other than cost)	Mail order, advise 3-mo refills
Regimen complexity	Simplify regimen (change to daily long-acting formulation, delete unnecessary/dangerous medications, suggest change to combination pills)
Beliefs about medications/condition	Education, motivational interviewing, medication action plan
Organizational difficulties	Pill boxes, other behavioral strategies

## Methods

The study protocol was reviewed and approved by the UCLA Institutional Review Board. Informed consent was not required per institutional policy for a quality improvement study. This study followed the Standards for Quality Improvement Reporting Excellence (SQUIRE) reporting guideline for quality improvement studies.

### Setting

The service area for Ronald Reagan UCLA Medical Center includes 18 cities/communities in Los Angeles County, California. The service area covers 656 039 people (69.7% aged 18-64 years and 14.3% aged ≥65 years). Roughly 16.5% of the residents of the service area are Hispanic. Spanish is spoken at home among 13.3% of the service area population.^14^

### Study Design

In this quality improvement study, we obtained EHR data for all patients in the exposure and usual care groups. The abstracted data included medical encounter types, demographics, diagnoses, vital signs, laboratory test results, prescription medications, and health insurance coverage variables.

The exposure group included adults with any instance of *International Classification of Diseases, Ninth Revision*/*International Statistical Classification of Diseases and Related Health Problems, Tenth Revision (ICD-9/10)* diagnosis code for T2D, self-reported Hispanic ethnicity entered into the EHR by patients, and age 18 years or older who had at least 1 face-to-face visit with a UCMyRx clinical pharmacist during the study window (March 2, 2013-December 31, 2018). Additionally, the exposure population for the HbA_1c_ analyses was limited to adults who had an HbA_1c_ concentration of 8% or higher, at least once between 365 days before and 14 days after the UCMyRx visit and a follow-up HbA_1c_ measure within 120 to 365 days of the visit. The SBP population was limited to adults who had an SBP of 140 mm Hg or higher, at least once between 365 days before and 14 days after the UCMyRx visit, and a follow-up SBP measure within 120 to 450 days after the visit. The longer duration for the SBP measure, relative to the HbA_1c_ measure, allowed the time to obtain 3 separate SBP measures and calculate the average of these measures, increasing the validity of the SBP results. The index date for the exposure population was the date of the first UCMyRx visit. The usual care group was drawn from all UCLA patients, with any instance of *ICD-9/10* diagnosis code for T2D, reporting Hispanic ethnicity in the EHR, who were aged 18 years or older and had at least 2 visits to 1 or more UCLA primary care clinics, 2 or more years apart, during the study window. This additional 2-visit criteria for the usual care group was necessary to allow for the generation of an intervening random index date to which the outcome measurement windows could be applied. Usual care patients were drawn from clinics both with and without a UCMyRx pharmacist; however, they did not have a visit with a UCMyRx pharmacist.

Our primary outcomes were pre– to post–index changes in HbA_1c_ and SBP measures. The pre-index HbA_1c_ concentration was the closest value to the index date with a window of 365 days before the index date and 14 days after. The pre-index SBP was the mean of the 3 values closest to the index date with a 365-day window before and a 14-day window after. The post-index HbA_1c_ concentration was the closest value to 180 days after the index date with a window of 120 to 365 days after the index date. The post-index SBP was the mean of the 3 values closest to 365 days after the index date with a window of 120 to 450 days after the index date. Systolic blood pressure was measured using Welch Allyn automated blood pressure cuffs. Concentration of HbA_1c_ was measured using high-performance liquid chromatography.

Since it is not possible to randomize patients to the UCMyRx program, we use propensity score matching to create comparable cohorts of UCMyRx-exposed and usual care patients.^15^ Logistic regression models were used to generate propensity scores. Variable choices for the propensity scores were informed by the extant literature and included pre-index or baseline (HbA_1c_ and SBP measures, age, sex, language preference (English vs non-English), Charlson comorbidity index (CCI), diabetes severity index (DSI) (a count of the following conditions: retinopathy, nephropathy, neuropathy, cardiovascular disease, or diabetes-related hospitalization),^16^ presence of serious mental illness (bipolar disorder, schizophrenia, major depression), body mass index (calculated as weight in kilograms divided by height in meters squared) category (<18.5, 18.5-24.9, 25.0-29.9, and ≥30), smoking status, having seen an endocrinologist (yes/no), number of diabetes medications, total number of prescription medications, and health insurance status (private, Medicare, Medicaid, Medicaid plus Medicare).^1,17^ Each UCMyRx patient was matched to 2 comparable usual care patients using the nearest neighbor matching propensity score matching method.^18^ Separate propensity score matching was done for each outcome.

### Statistical Analysis

Statistical software R, version 4.0.3 (R Foundation for Statistical Computing) was used for analyses.^19^ The unit of analysis was the patient. We calculated descriptive statistics for all variables in the models, across treatment status, using *t* test and χ^2^ test to compare continuous and dichotomous/categorical variables, respectively, with *P* < .05 considered statistically significant. To evaluate the association of the UCMyRx program with HbA_1c_ concentration and SBP, we performed difference-in-differences (DID) analyses.^15^ The DID study design is particularly well suited to assess the associations of the UCMyRx intervention given that it is able to remove the influence of other potential interventions, such as a systemwide diabetes care quality improvement initiative, provided that both the UCMyRx-exposed and comparison groups were exposed to the intervention and both groups were affected by the intervention in the same way. The use of propensity score matching helped ensure that the UCMyRx-exposed and comparison groups were balanced on observable factors that may influence how they would respond to a given intervention. We used linear mixed-effects models that included an indicator for time (post-index vs pre-index) that was coded as “1” if the observation was from the post-index period and coded “0” otherwise, an indicator for treatment status (UCMyRx-exposed vs usual care) that was coded as “1” if the observation was from the UCMyRx group and coded as “0” otherwise, and the interaction between time and group (primary predictor), among the matched samples. The models also included random effects to take into account data clustering within each pair of matched UCMyRx-exposed and usual care patients and data clustering within each patient. To assess for UCMyRx point estimate differences, across language preference, we ran stratified analyses, across primary language preference (English vs non-English language preference). Furthermore, to assess for differential associations between UCMyRx exposure and risk factors contingent on the number of contacts with a UCMyRx pharmacist, we ran analyses comparing individuals with face-to-face contacts and total contacts (face-to-face visits, telephone calls, and emails) above the median with those at the median or below, by incorporating an interaction between time and an indicator coded as “1” if the number of contacts was above the median and coded “0” otherwise, in a model that was limited to UCMyRx-exposed Hispanic patients. Lastly, we repeated our main analyses with UCMyRx-exposed White patients as the comparison group instead of Hispanic patients receiving usual care. For these analyses, the covariates in the propensity score–matching model remained the same, but due to sample size limitations, we were only able to match 1:1 rather than 2:1.

## Results

Of the 931 unique patients with T2D analyzed, the mean (SD) age was 64 (14.1) years, 552 (59.3%) were female, and 463 (49.7%) had an English language preference. Our postmatching sample sizes for the main HbA_1c_ and SBP outcomes were 396 and 795, respectively (Figure). Some patients (260) were eligible for both the HbA_1c_ and SBP samples. Descriptive statistics for each of the unmatched and matched analytic samples are shown in eTable 1 (HbA_1c_ sample) and eTable 2 (SBP sample) in Supplement 1. Prior to matching, there were statistically significant differences across the UCMyRx-exposed and comparison group in the HbA_1c_ subsample. The UCMyRx-exposed HbA_1c_ subsample had a larger proportion of individuals 65 years or older (49% vs 34%) and a higher proportion of female individuals (59% vs 48%), were less likely to preferer English (48% vs 72%), had a lower proportion of individuals with private insurance (44% vs 63%) and a higher proportion of individuals with Medicare plus Medicaid insurance (dual enrollment) (41% vs 14%), were receiving more total medications (11 vs 9), and had a higher CCI (7.9 vs 5.1), a higher DSI (6.0 vs 3.7), and a higher mean baseline HbA_1c_ concentration (9.2% vs 8.3%). Postmatching, statistically significant differences remained across the treatment and comparison groups for dual-enrollment status and CCI score. With respect to the SBP subsample, there were also statistically significant differences across the UCMyRx-exposed and comparison groups. The UCMyRx-exposed SBP subsample had a larger proportion of individuals 65 years or older (56% vs 46%), were less likely to preferer English (46% vs 69%), had a lower proportion of individuals with private insurance (45% vs 68%) and a higher proportion of individuals with dual enrollment (36% vs 17%), were receiving more total medications (10 vs 8), and had a higher CCI (7.1 vs 4.9), a higher DSI (5.2 vs 3.2), and a higher mean baseline HbA_1c_ concentration (7.7% vs 7.0%). Postmatching, no statistically significant differences across the UCMyRx-exposed and usual care SBP subsamples remained. Descriptive statistics for each of the unmatched and matched analytic subsamples used in our language-stratified and White comparison group sensitivity analyses are shown in eTables 3-6 and 7-8 in Supplement 1, respectively. Over the time period covered by the study window, the mean (SD) number of face-to-face visits with a clinical pharmacist for the main HbA_1c_ and SBP samples were 4.65 (7.04) and 3.89 (5.82), respectively. The mean (SD) number of total contacts (face-to-face visits, telephone, and email) with the clinical pharmacist for the HbA_1c_ and SBP samples were 7.14 (9.91) and 5.98 (8.17), respectively.

**Figure.**
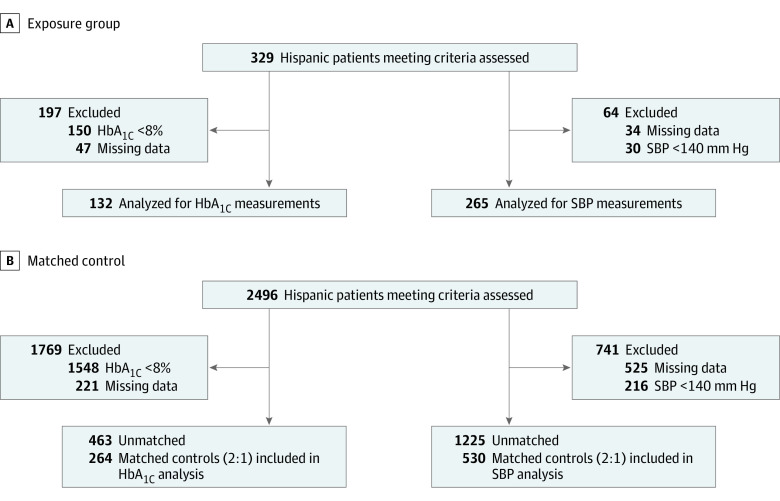
Study Flow Diagram HbA_1c_ indicates hemoglobin A_1c_; SBP, systolic blood pressure.

The results of our adjusted main analyses are shown in Table 2. Having at least 1 clinical pharmacist visit was associated with a significant reduction in HbA_1c_ concentration (β = −0.46%; 95% CI, (−0.84% to −0.07%; *P* = .02) and no change in SBP (β = −1.71 mm Hg; 95% CI, −4.01 to 0.58 mm Hg; *P* = .14) among the HbA_1c_ and SBP samples, respectively. In language-stratified analyses (Table 3), we found a significant negative association of UCMyRx exposure with HbA_1c_ concentration among the HbA_1c_ subsample with an English language preference (β = −0.59%; 95% CI, −1.13% to −0.06%; *P* = .03), but no significant trend toward a negative association among the subsample with a non-English language preference (β = −0.38%; 95% CI, −0.92% to 0.17%; *P* = .58). Exposure to UCMyRx was not associated with SBP among both language-preference subgroups (β = −2.74 mm Hg; 95% CI, −5.86 to 0.39 mm Hg; *P* = .08) and (β = −0.53 mm Hg; 95% CI, −3.91 to 2.86 mm Hg; *P* = .76) for Non-English and English language preference, respectively. We did not find a significant association between UCMyRx exposure and HbA_1c_ concentration among individuals with more than the median number of face-to-face visits (β = −0.18%; 95% CI, −0.79% to 0.43%; *P* = .56) and total contacts (β = −0.19%; 95% CI, −0.81% to 0.42%; *P* = .53), respectively. Likewise, there was no significant association between SBP and more face-to-face visits (β = 0.47%; 95% CI, −3.48% to 4.41%; *P* = .82) or total contacts (β = 0.37%; 95% CI, −3.58% to 4.34%; *P* = .85) (not shown). In our sensitivity analyses with the White comparison groups, we found no significant difference in HbA_1c_ concentration (β = −0.17%; 95% CI, −0.62% to 0.27%; *P* = .44) or SBP (β = −0.04 mm Hg; 95% CI, −2.61 to 2.53 mm Hg; *P* = .98) over time across UCMyRx-exposed Hispanic and White patients (Table 4).

**Table 2.** Association of UCMyRx Exposure With Risk Factor Change (All Patients)^a^

	HbA_1c_^b^		SBP^c^	
	β (95% CI), %	*P* value	β (95% CI), mm Hg	*P* value
UCMyRx × time	−0.46 (−0.84 to −0.07)	.02	−1.71 (−4.01 to 0.58)	.14

Abbreviations: HbA_1c_, hemoglobin A_1c_; SBP, systolic blood pressure.

^a^ The β coefficients were generated using difference-in-differences analysis with linear mixed-effects models that included fixed effects for time (pre– vs post–index date), group (UCMyRx-exposed vs usual care) and the interaction between time and group among the propensity score–matched samples. Propensity scores were generated using logistic regression models that included pre-index (baseline) HbA_1c_ and SBP measures, age, sex, language preference (English vs non-English), body mass index category, smoking status, Charlson comorbidity index, diabetes severity index, presence of serious mental illness (bipolar disorder, schizophrenia, major depression), having seen an endocrinologist 1 or more times, number of diabetes medications, total number of medications, and health insurance status. Each UCMyRx-exposed patient was matched to 2 usual care patients using a nearest neighbor matching approach.

^b^ N = 396.

^c^ N = 795.

**Table 3.** Association of UCMyRx Exposure With Risk Factor Change (Language Stratified)^a^

	HbA_1c_^b^		SBP^c^	
	β (95% CI), %	*P* value	β (95% CI), mm Hg	*P* value
English language preference	−0.59 (−1.13 to −0.06)	.03	−0.53 (−3.91 to 2.86)	.76
Non-English language preference	−0.38 (−0.92 to 0.17)	.17	−2.74 (−5.86 to 0.39)	.09

Abbreviations: HbA_1c_, hemoglobin A_1c_; SBP, systolic blood pressure.

^a^ Models were stratified by language preference (English vs non-English preference). The beta coefficients were generated using difference-in-differences analysis with linear mixed-effects models that included fixed effects for time (pre– vs post–index date), group (UCMyRx-exposed vs usual care) and the interaction between time and group among the propensity score–matched samples. Propensity scores were generated using logistic regression models that included pre-index (baseline) HbA_1c_ and SBP measures, age, sex, language preference (English vs non-English), body mass index category, smoking status, Charlson comorbidity index, diabetes severity index, presence of serious mental illness (bipolar disorder, schizophrenia, major depression), having seen an endocrinologist 1 or more times, number of diabetes medications, total number of medications, and health insurance status. Individuals were not rematched to retain the same patients in the main and sensitivity analyses.

^b^ N = 209 for English preference, and n = 187 non-English preference.

^c^ N = 363 for English preference, and n = 432 for non-English preference.

**Table 4.** Association of UCMyRx Exposure With Risk Factor Change (Hispanic vs Non-Hispanic White Exposed Patients)^a^

	HbA_1c_^b^		SBP^c^	
	β (95% CI), %	*P* value	β (95% CI), mm Hg	*P* value
UCMyRx × time	−0.17 (−0.62 to 0.27)	.44	−0.04 (−2.61 to 2.53)	.98

Abbreviations: HbA_1c_, hemoglobin A_1c_; SBP, systolic blood pressure.

^a^ The β coefficients were generated using difference-in-differences analysis with linear mixed-effects models that included fixed effects for time (pre– vs post–index date), group (Hispanic vs non-Hispanic White) and the interaction between time and group among the propensity score–matched samples. Propensity scores were generated using logistic regression models that included pre-index (baseline) HbA_1c_ and SBP measures, age, sex, language preference (English vs non-English), body mass index category, smoking status, Charlson comorbidity index, diabetes severity index, presence of serious mental illness (bipolar disorder, schizophrenia, major depression), having seen an endocrinologist 1 or more times, number of diabetes medications, total number of medications, and health insurance status. Each UCMyRx-exposed Hispanic patient was matched to 1 UCMyRx-exposed White patient using a nearest neighbor matching approach.

^b^ N = 264.

^c^ N = 530.

## Discussion

In a study of an intervention that embeds a clinical pharmacist into primary care practices to conduct medication reconciliation/simplification, introduce personally tailored strategies for medication adherence, augment diabetes self-management behavior, and increase guideline-concordant treatment intensification on HbA_1c_ concentration and SBP, we found that having at least 1 face-to-face visit with a clinical pharmacist was associated with a statistically significant reduction in HbA_1c_ concentration but not SBP among Hispanic patients. Our sensitivity analyses based on visit frequency suggested a negative association between HbA_1c_ and UCMyRx exposure with a single UCMyRx pharmacist visit.

In sensitivity analyses among the HbA_1c_ sample, we found that reductions in HbA_1c_ concentration observed among UCMyRx-exposed patients were primarily driven by the association among patients with an English language preference. Relative to patients in the HbA_1c_ subsample with a non-English language preference, those with an English language preference appeared to be younger, had a lower CCI and a lower DSI, and were more likely to have private health insurance (eTables 3 and 4 in Supplement 1). Current guidelines for T2D management suggest more aggressive HbA_1c_ control among younger individuals with lower levels of comorbidity, which may translate into more aggressive HbA_1c_ goals among the UCMyRx-exposed patients with an English language preference, relative to those with a non-English language preference.^20^ We did not find a significant association between UCMyRx exposure and SBP in any of the analyses. This may be attributable to the relatively low baseline SBPs among the SBP subsamples (135-136 mm Hg). Furthermore, we did not find differences in the association between UCMyRx exposure and HbA_1c_ concentration across Hispanic and White patients. This finding suggests that UCMyRx may have similar benefit across ethnic groups.

An HbA_1c_ reduction of 0.46% is consistent with what some studies have found for insulin initiation.^21^ Economic models have predicted that a 0.4% decrease in HbA_1c_ concentration would significantly reduce microvascular and macrovascular complications among patients with diabetes, over 25 years, taking into account age, sex, risk factors, and preexisting complications.^22,23^ The 0.59% mean HbA_1c_ reduction observed among UCMyRx-exposed patients with an English language preference, relative to usual care, is on the order of what has been observed for some diabetes medications.^24^ With respect to what may be underlying this HbA_1c_ change, an internal review of all the patients served by the UCMyRx intervention (not restricted to Hispanic individuals) conducted in the first 3 years of the program found that 24% had an inaccurate medication list, 37% were not taking medications as directed, and 46% were nonadherent based on the results of the standardized survey. The most common reasons for nonadherence were intolerable adverse effects, memory issues, out-of-pocket cost concerns, and beliefs regarding medications and/or conditions. Nonetheless, UCMyRx exposure was associated with clinically meaningful changes in HbA_1c_ concentration among Hispanic primary care patients with T2D, particularly those with an English language preference.

Given the potential of pharmacist-led interventions like UCMyRx to help improve outcomes in T2D while simultaneously supporting PCPs, it is important to facilitate their broader uptake.^13^ Prerequisites for wider dissemination of such interventions include a broader scope of practice for pharmacists.^25^ California law SB 493, which was signed into law in 2013, designated pharmacists as “healthcare providers” who are authorized to provide health care services, a designation that allows pharmacists to participate in multidisciplinary review of patient progress, including appropriate access to medical records, and provide consultation, training, and education to patients about drug therapy, disease management, and disease prevention.^26^ Lastly, as of June 2014, in alignment with California SB 493, UCMyRx pharmacists began billing directly for their consultation services, an important change to promote UCMyRx sustainability.

### Limitations

The results of this study must be viewed in the context of limitations. The DID with propensity score–matching study design cannot account for differences in nonobservable factors. We cannot assess how differences in UCMyRx access point influence the association between UCMyRx exposure and study outcomes. Visit count stipulations for the comparison but not the treatment groups may have biased the results toward the null. Furthermore, we lack detailed data on the content of the pharmacist-led interventions. Lastly, these results pertain to Hispanic patients with T2D being seen in an academic health center located in southern California between 2013 and 2018. These data years may not reflect current patterns of clinical practice. Despite the limitations, this study makes a contribution to the evidence base of strategies to improve diabetes outcomes.

## Conclusions

In this quality improvement study, exposure to a pharmacist-led intervention was associated with a reduction in HbA_1c_ concentration among Hispanic patients with T2D. This association did not vary across ethnicity. The findings of this study suggest that pharmacist-led intervention may be a strategy to improve diabetes outcomes, irrespective of ethnicity.

## Notes

Supplement 1. **eTable 1.** Descriptive Statistics by Treatment Status for the HbA1c Sample (Unmatched and Matched) **eTable 2.** Descriptive Statistics by Treatment Status for the Systolic Blood Pressure Sample (Unmatched and Matched) **eTable 3.** Descriptive Statistics by Treatment Status for the HbA1c Sample English Speaking (Unmatched and Matched) **eTable 4.** Descriptive Statistics by Treatment Status for the HbA1c Sample Non-English Speaking (Unmatched and Matched) **eTable 5.** Descriptive Statistics by Treatment Status for the Systolic Blood Pressure Sample English Speaking (Unmatched and Matched) **eTable 6.** Descriptive Statistics by Treatment Status for the Systolic Blood Pressure Sample Non-English Speaking (Unmatched and Matched) **eTable 7.** Descriptive Statistics by Treatment Status for HbA1c Sample Exposed Hispanic vs Exposed Non-Hispanic White (Unmatched and Matched) **eTable 8.** Descriptive Statistics by Treatment Status for the Systolic Blood Pressure Sample Exposed Hispanic vs Exposed Non-Hispanic White (Unmatched and Matched)

Supplement 2.
Data Sharing Statement
