# Supplementary material for: Pharmacist-Led Diabetes Control Intervention and Health Outcomes in Hispanic Patients With Diabetes
Source: JAMA Netw Open. 2023 Sep 28;6(9):e2335409. doi: 10.1001/jamanetworkopen.2023.35409 (PMC10539985; doi:10.1001/jamanetworkopen.2023.35409)
Supplement: Supplement 1. — eTable 1. Descriptive Statistics by Treatment Status for the HbA1c Sample (Unmatched and Matched) eTable 2. Descriptive Statistics by Treatment Status for the Systolic Blood Pressure Sample (Unmatched and Matched) eTable 3. Descriptive Statistics by Treatment Status for the HbA1c Sample English Speaking (Unmatched and Matched) eTable 4. Descriptive Statistics by Treatment Status for the HbA1c Sample Non-English Speaking (Unmatched and Matched) eTable 5. Descriptive Statistics by Treatment Status for the Systolic Blood Pressure Sample English Speaking (Unmatched and Matched) eTable 6. Descriptive Statistics by Treatment Status for the Systolic Blood Pressure Sample Non-English Speaking (Unmatched and Matched) eTable 7. Descriptive Statistics by Treatment Status for HbA1c Sample Exposed Hispanic vs Exposed Non-Hispanic White (Unmatched and Matched) eTable 8. Descriptive Statistics by Treatment Status for the Systolic Blood Pressure Sample Exposed Hispanic vs Exposed Non-Hispanic White (Unmatched and Matched) [file jamanetwopen-e2335409-s001.pdf]

Supplemental Online Content

Narain KC, Moreno G, Bell DS, et al. Pharmacist-led diabetes control intervention and health outcomes in Hispanic patients with diabetes. *JAMA Netw Open.* 2023;6(9):e2335409. doi:10.1001/jamanetworkopen.2023.35409

eTable 1. Descriptive Statistics by Treatment Status for the HbA1c Sample (Unmatched and Matched)

eTable 2. Descriptive Statistics by Treatment Status for the Systolic Blood Pressure Sample (Unmatched and Matched)

eTable 3. Descriptive Statistics by Treatment Status for the HbA1c Sample English Speaking (Unmatched and Matched)

eTable 4. Descriptive Statistics by Treatment Status for the HbA1c Sample Non-English Speaking (Unmatched and Matched)

eTable 5. Descriptive Statistics by Treatment Status for the Systolic Blood Pressure Sample English Speaking (Unmatched and Matched)

eTable 6. Descriptive Statistics by Treatment Status for the Systolic Blood Pressure Sample Non-English Speaking (Unmatched and Matched)

eTable 7. Descriptive Statistics by Treatment Status for HbA1c Sample Exposed Hispanic vs Exposed Non-Hispanic White (Unmatched and Matched)

eTable 8. Descriptive Statistics by Treatment Status for the Systolic Blood Pressure Sample Exposed Hispanic vs Exposed Non-Hispanic White (Unmatched and Matched)

This supplemental material has been provided by the authors to give readers additional information about their work.

© 2023 Narain KC et al. *JAMA Network Open.*

eTable 1. Descriptive Statistics by Treatment Status for the HbA1c Sample (Unmatched and Matched)					
**Covariates[1]**	**Source Population for Usual Care n=727**	***p*-value**	**UCMyRx n=132**	***p*-value**	**Matched Comparison n=264**
Age in years		0.005		0.85	
18-44	116 (16)		12 (9.1)		31 (11.7)
45-64	359 (49.4)		54 (40.9)		103 (39)
65-74	169 (23.2)		47 (35.6)		89 (33.7)
75+	83 (11.4)		19 (14.4)		41 (15.5)
Gender		0.03		1	
Female	355 (48.8)		79 (59.8)		159 (60.2)
Male	372 (51.2)		53 (40.2)		105 (39.8)
Smoking		1		0.78	
Non-Smoker	697 (95.9)		126 (95.6)		255 (96.6)
Currently Smoking	30 (4.1)		6 (4.5)		9 (3.4)
English Language		<.001		0.27	
No	198 (27.2)		68 (51.5)		119 (45.076)
Yes	529 (72.8)		64 (48.5)		145 (54.924)
BMI		0.94		0.76	
<18.5	1 (0.1)		0 (0)		0 (0)
18.5-24.9	102 (14)		17 (12.9)		41 (15.53)
25-29.9	232 (31.9)		41 (31.1)		82 (31.061)
>=30	392 (53.9)		74 (56.1)		141 (53.409)
Insurance		<.001		0.40	
Medicaid	31 (4.3)		11 (8.3)		15 (5.7)
Medicare	232 (31.9)		62 (47)		115 (43.6)
Private[1]	464 (63.8)		59 (44.7)		134 (50.8)
Medicare-Medicaid		<.001		0.05	
No	619 (85.1)		77 (58.3)		182 (68.9)
Yes	108 (14.9)		55 (41.667)		82 (31.1)
Any Endocrinology Visit		0.57		0.73	
No	189 (25.997)		38 (28.788)		82 (31.1)
Yes	538 (74.003)		94 (71.212)		182 (68.9)
Number Medications - [Mean(SD)]	9.3 (5.1)	<.001	11.417 (6)	0.36	10.8 (5.3)
Number Diabetes Medications - [Mean(SD)]	1.3(0.9)	0.20	1.2 (1.1)	0.39	1.3 (1)
Charlson Comorbidity Index [Mean(SD)]	5.2 (3.4)	<.001	7.9 (4)	0.05	7.1 (3.8)
Diabetes Severity Index [Mean(SD)]	3.7 (2.9)	<.001	6 (3.3)	0.07	5.4 (3.1)
Any Serious Mental Illness		<.001		0.41	
No	447 (61.5)		50 (37.9)		113 (42.8)
Yes	280 (38.5)		82 (62.1)		151 (57.2)
HbA1c [Mean(SD)]	8.3 (1.8)	<.001	9.2 (2.2)	0.10	8.9(2.1)
SBP [Mean(SD)]	132.6 (15.1)	0.52	134 (16.6)	0.97	133.7(16.7)

1. All covariates are measured during the pre-index (baseline period). Propensity scores were generated using logistic regression models that included pre-index (HbA1c and SBP levels, age, gender, language preference, BMI category, smoking status, Charlson Co-morbidity index, Diabetes Severity Index, presence of serious mental illness (bipolar disorder, schizophrenia, major depression), having seen an endocrinologist ≥ 1 time, number of diabetes medications, total number of medications and health insurance status. Each UCMyRx-exposed patient was matched to two usual care patients using a Nearest Neighbor Matching approach. Bivariates were generated using t-test and Chi-squared test for continuous and categorical/dichotomous variables, respectively.

© 2023 Narain KC et al. *JAMA Network Open.*

eTable 2. Descriptive Statistics by Treatment Status for the Systolic Blood Pressure Sample (Unmatched and Matched)					
Covariates[1]	Source Population for Usual Care n=1755	*p*-value	UCMyRx n=265	*p*-value	Matched Comparison n=530
Age in years		0.002		0.94	
18-44	198 (11.3)		18 (6.8)		39 (7.358)
45-64	747 (42.6)		96 (36.2)		181 (34.151)
65-74	493 (28.1)		83 (31.3)		167 (31.509)
75+	317 (18.1)		68 (25.7)		143 (26.981)
Gender		0.13		0.52	
Female	982 (56)		162 (61.1)		310 (58.5)
Male	773 (44)		103 (38.9)		220 (41.5)
Smoking		0.96		0.84	
Non-Smoker	1687 (96.1)		254 (95.8)		511 (96.4)
Currently Smoking	68 (3.9)		11 (4.2)		19 (3.6)
English Language		<.001		0.82	
No	530 (30.2)		142 (53.6)		290 (54.7)
Yes	1225 (69.8)		123 (46.4)		240 (45.3)
BMI		0.16		1	
<18.5	5 (0.3)		3 (1.1)		5 (0.9)
18.5-24.9	262 (14.9)		40 (15.1)		80 (15.1)
25-29.9	600 (34.2)		82 (30.9)		164 (30.9)
>=30	888 (50.6)		140 (52.8)		281 (53)
Insurance		<.001		0.97	
Medicaid	60 (3.4)		16 (6)		30 (5.7)
Medicare	661 (37.7)		128 (48.3)		254 (47.9)
Private[1]	1034 (58.9)		121 (45.7)		246 (46.4)
Medicare-Medicaid		<.001		0.58	
No	1440 (82.1)		167 (63)		346 (65.2)
Yes	315 (17.9)		98 (37)		184 (34.7)
Any Endocrinology Visit		0.06		0.41	
No	902 (51.4)		119 (44.9)		256 (48.3)
Yes	853 (48.6)		146 (55.1)		274 (51.7)
Number Medications - [Mean(SD)]	8.21 (5.4)	<.001	10.521 (5.6)	0.30	10.2 (5.7)
Number Diabetes Medications - [Mean(SD)]	0.8 (0.9)	0.88	0.8(1)	0.55	0.8 (0.9)
Charlson Comorbidity Index [Mean(SD)]	4.9 (3.4)	<.001	7.2(3.9)	0.38	6.9 (3.9)
Diabetes Severity Index [Mean(SD)]	3.3 (2.8)	<.001	5.3(3.2)	0.43	5 (3)
Any Serious Mental Illness		<.001		0.56	
No	1063 (60.6)		112 (42.3)		237 (44.7)
Yes	692 (39.4)		153 (57.7)		293 (55.3)
HbA1c [Mean(SD)]	7 (1.6)	<.001	7.7 (2.1)	0.42	7.5(1.9)
SBP [Mean(SD)]	135.3 (13.9)	0.95	136 (16.8)	0.44	136.3 (14.7)

1. All covariates are measured during the pre-index (baseline period). Propensity scores were generated using logistic regression models that included pre-index (HbA1c and SBP levels, age, gender, language preference, BMI category, smoking status, Charlson Co-morbidity index, Diabetes Severity Index, presence of serious mental illness (bipolar disorder, schizophrenia, major depression), having seen an endocrinologist ≥ 1 time, number of diabetes medications, total number of medications and health insurance status. Each UCMyRx-exposed patient was matched to two usual care patients using a Nearest Neighbor Matching approach. Bivariates were generated using t-test and Chi-squared test for continuous and categorical/dichotomous variables, respectively.

© 2023 Narain KC et al. *JAMA Network Open.*

eTable 3. Descriptive Statistics by Treatment Status for the HbA1c Sample English Speaking (Unmatched and Matched)					
**Covariates[1]**	**Source Population for Usual Care n=529**	***p*-value**	**UCMyRx n=64**	***p*-value**	**Matched Comparison n=145**
Age in years		0.71		0.80	
18-44	107 (20.2)		11 (17.2)		28 (19.3)
45-64	291 (55)		34 (53.1)		71 (49)
65-74	99 (18.7)		13 (20.3)		36 (24.8)
75+	32 (6)		6 (9.4)		10 (6.9)
Gender		0.15		0.85	
Female	234 (44.2)		35 (54.7)		83 (57.2)
Male	295 (55.8)		29 (45.3)		62 (42.8)
Smoking		1		1	
Non-Smoker	502 (94.9)		61 (95.3)		137 (94.5)
Currently Smoking	27 (5.1)		3 (4.7)		8 (5.5)
BMI		0.94		0.81	
<18.5	1 (0.2)		0 (0)		0 (0)
18.5-24.9	61 (11.5)		6 (9.4)		18 (12.4)
25-29.9	155 (29.3)		19 (29.7)		40 (27.6)
>=30	312 (59)		39 (60.9)		87 (60)
Insurance		0.01		0.58	
Medicaid	21 (4)		7 (10.9)		10 (6.9)
Medicare	138 (26.1)		23 (35.9)		51 (35.2)
Private[1]	370 (69.9)		34 (53.1)		84 (57.9)
Medicare-Medicaid		<.001		0.0617	
No	494 (93.4)		46 (71.9)		122 (84.1)
Yes	35 (6.6)		18 (28.1)		23 (15.9)
Any Endocrinology Visit		0.02		0.14	
No	130 (24.6)		25 (39.1)		40 (27.6)
Yes	399 (75.4)		39 (60.9)		105 (72.4)
Number Medications - [Mean(SD)]	8.7 (4.9)	<.001	11.5 (6.2)	0.16	10.1 (5.3)
Number Diabetes Medications - [Mean(SD)]	1.3 (0.9)	0.60	1.2 (1.1)	0.78	1.3(1)
Charlson Comorbidity Index [Mean(SD)]	4.8 (3.3)	<.001	7.4 (3.8)	0.17	6.9 (4.2)
Diabetes Severity Index [Mean(SD)]	3.3 (2.8)	<.001	5.7 (3.7)	0.08	4.7 (3.1)
Any Serious Mental Illness		<.001		0.90	
No	335 (63.3)		23 (35.938)		55 (37.931)
Yes	194 (36.7)		41 (64.062)		90 (62.069)
HbA1C [Mean(SD)]	8.4 (1.9)	0.005	9.1 (2)	0.50	9.4 (2.4)
SBP [Mean(SD)]	131.7 (14.3)	0.33	134.4(17.2)	0.26	131.2(16)

1. All covariates are measured during the pre-index (baseline period). Propensity scores were generated using logistic regression models that included pre-index (HbA1c and SBP levels, age, gender, language preference, BMI category, smoking status, Charlson Co-morbidity index, Diabetes Severity Index, presence of serious mental illness (bipolar disorder, schizophrenia, major depression), having seen an endocrinologist ≥ 1 time, number of diabetes medications, total number of medications and health insurance status. Each UCMyRx-exposed patient was matched to two usual care patients using a Nearest Neighbor Matching approach. Bivariates were generated using t-test and Chi-squared test for continuous and categorical/dichotomous variables, respectively.

© 2023 Narain KC et al. *JAMA Network Open.*

eTable 4. Descriptive Statistics by Treatment Status for the HbA1c Sample Non-English Speaking (Unmatched and Matched)					
Covariates[1]	Source Population for Usual Care n=198	*p*-value	UCMyRx n=68	*p*-value	Matched Comparison n=119
Age in years		0.15		0.69	
18-44	9 (4.5)		1 (1.5)		3 (2.5)
45-64	68 (34.3)		20 (29.4)		32 (26.9)
65-74	70 (35.4)		34 (50)		53 (44.5)
75+	51 (25.8)		13 (19.1)		31 (26.1)
Gender		0.70		1	
Female	121 (61.1)		44 (64.7)		76 (63.9)
Male	77 (38.9)		24 (35.3)		43 (36.1)
Smoking		0.36		0.27	
Non-Smoker	195 (98.5)		65 (95.6)		118 (99.2)
Currently Smoking	3 (1.5)		3 (4.4)		1 (0.8)
BMI		0.28		0.71	
<18.5	0 (0)		0 (0)		0 (0)
18.5-24.9	41 (20.7)		11 (16.2)		23 (19.3)
25-29.9	77 (38.9)		22 (32.4)		42 (35.3)
>=30	80 (40.4)		35 (51.5)		54 (45.4)
Insurance		0.31		0.72	
Medicaid	10 (5.1)		4 (5.9)		5 (4.2)
Medicare	94 (47.5)		39 (57.4)		64 (53.8)
Private[1]	94 (47.5)		25 (36.8)		50 (42)
Medicare-Medicaid		0.02		0.63	
No	125 (63.1)		31 (45.6)		60 (50.4)
Yes	73 (36.9)		37 (54.4)		59 (49.6)
Any Endocrinology Visit		0.12		0.03	
No	59 (29.8)		13 (19.1)		42 (35.3)
Yes	139 (70.2)		55 (80.9)		77 (64.7)
Number Medications - [Mean(SD)]	10.8 (5.3)	0.28	11.4 (5.8)	0.78	11.7 (5.1)
Number Diabetes Medications - [Mean(SD)]	1.3 (0.9)	0.20	1.2 (1.2)	0.34	1.3 (0.9)
Charlson Comorbidity Index [Mean(SD)]	6.3 (3.3)	<.001	8.4 (4.2)	0.19	7.4(3.3)
Diabetes Severity Index [Mean(SD)]	5 (3)	0.002	6.3 (3)	0.74	6.2 (2.8)
Any Serious Mental Illness		0.02		0.30	
No	112 (56.6)		27 (39.7)		58 (48.7)
Yes	86 (43.4)		41 (60.3)		61 (51.3)
HbA1c [Mean(SD)]	8.1 (1.5)	<.001	9.3 (2.3)	0.002	8.2 (1.6)
SBP [Mean(SD)]	135.3 (16.6)	0.41	133.6 (16)	0.16	136.7 (17)

1. All covariates are measured during the pre-index (baseline period). Propensity scores were generated using logistic regression models that included pre-index (HbA1c and SBP levels, age, gender, language preference, BMI category, smoking status, Charlson Co-morbidity index, Diabetes Severity Index, presence of serious mental illness (bipolar disorder, schizophrenia, major depression), having seen an endocrinologist ≥ 1 time, number of diabetes medications, total number of medications and health insurance status. Each UCMyRx-exposed patient was matched to two usual care patients using a Nearest Neighbor Matching approach. Bivariates were generated using t-test and Chi-squared test for continuous and categorical/dichotomous variables, respectively.

© 2023 Narain KC et al. *JAMA Network Open.*

eTable 5. Descriptive Statistics by Treatment Status for the Systolic Blood Pressure Sample English Speaking (Unmatched and Matched)					
**Covariates[1]**	**Source Population for Usual Care n=1225**	***p*-value**	**UCMyRx n=123**	***p*-value**	**Matched Comparison n=240**
Age in years		0.82		0.91	
18-44	187 (15.3)		17 (13.8)		34 (14.2)
45-64	591 (48.2)		57 (46.3)		108 (45)
65-74	292 (23.8)		30 (24.4)		54 (22.5)
75+	155 (12.7)		19 (15.4)		44 (18.3)
Gender		0.46		0.40	
Female	639 (52.2)		69 (56.1)		122 (50.8)
Male	586 (47.8)		54 (43.9)		118 (49.2)
Smoking		0.49		0.98	
Non-Smoker	1168 (95.3)		115 (93.5)		226 (94.2)
Currently Smoking	57 (4.7)		8 (6.5)		14 (5.8)
BMI		0.02		0.29	
<18.5	4 (0.3)		3 (2.4)		4 (1.7)
18.5-24.9	151 (12.3)		18 (14.6)		22 (9.2)
25-29.9	401 (32.7)		37 (30.1)		66 (27.5)
>=30	669 (54.6)		65 (52.8)		148 (61.7)
Insurance		0.02		0.90	
Medicaid	39 (3.2)		7 (5.7)		16 (6.7)
Medicare	388 (31.7)		51 (41.5)		95 (39.6)
Private[1]	798 (65.1)		65 (52.8)		129 (53.8)
Medicare-Medicaid		<.001		0.15	
No	1122 (91.6)		94 (76.4)		200 (83.3)
Yes	103 (8.4)		29 (23.6)		40 (16.7)
Any Endocrinology Visit		0.50		0.72	
No	612 (50)		57 (46.3)		105 (43.8)
Yes	613 (50)		66 (53.7)		135 (56.2)
Number Medications - [Mean(SD)]	7.8 (5.2)	<.001	10.016 (5.52)	0.56	10.3 (5.7)
Number Diabetes Medications - [Mean(SD)]	0.7 (0.8)	0.99	0.8(1)	0.76	0.8 (0.9)
Charlson Comorbidity Index [Mean(SD)]	4.6 (3.3)	<.001	6.6 (3.6)	0.81	6.7 (4.1)
Diabetes Severity Index [Mean(SD)]	2.9 (2.6)	<.001	5 (3.5)	0.98	4.9 (3)
Any Serious Mental Illness		<.001		0.76	
No	770 (62.9)		52 (42.3)		96 (40)
Yes	455 (37.1)		71 (57.7)		144 (60)
HbA1c [Mean(SD)]	7.1 (1.7)	<.001	7.8 (2.1)	0.71	7.8 (2.2)
SBP [Mean(SD)]	134.7 (13.9)	0.88	135.4(17.1)	0.99	134.7(14.7)

1. All covariates are measured during the pre-index (baseline period). Propensity scores were generated using logistic regression models that included pre-index (HbA1c and SBP levels, age, gender, language preference, BMI category, smoking status, Charlson Co-morbidity index, Diabetes Severity Index, presence of serious mental illness (bipolar disorder, schizophrenia, major depression), having seen an endocrinologist ≥ 1 time, number of diabetes medications, total number of medications and health insurance status. Each UCMyRx-exposed patient was matched to two usual care patients using a Nearest Neighbor Matching approach. Bivariates were generated using t-test and Chi-squared test for continuous and categorical/dichotomous variables, respectively.

© 2023 Narain KC et al. *JAMA Network Open.*

eTable 6. Descriptive Statistics by Treatment Status for the Systolic Blood Pressure Sample Non-English Speaking (Unmatched and Matched)					
**Covariates[1]**	**Source Population for Usual Care n=530**	***p*-value**	**UCMyRx n=142**	***p*-value**	**Matched Comparison n=290**
Age in years		0.59		0.81	
18-44	11 (2.1)		1 (0.7)		5 (1.7)
45-64	156 (29.4)		39 (27.5)		73 (25.2)
65-74	201 (37.9)		53 (37.3)		113 (39)
75+	162 (30.6)		49 (34.5)		99 (34.1)
Gender		0.94		0.98	
Female	343 (64.7)		93 (65.5)		188 (64.8)
Male	187 (35.3)		49 (34.5)		102 (35.2)
Smoking		1		1	
Non-Smoker	519 (97.9)		139 (97.9)		285 (98.3)
Currently Smoking	11 (2.1)		3 (2.1)		5 (1.7)
BMI		0.09		0.45	
<18.5	1 (0.2)		0 (0)		1 (0.3)
18.5-24.9	111 (20.9)		22 (15.5)		58 (20)
25-29.9	199 (37.5)		45 (31.7)		98 (33.8)
>=30	219 (41.3)		75 (52.8)		133 (45.9)
Insurance		0.33		0.80	
Medicaid	21 (4)		9 (6.3)		14 (4.8)
Medicare	273 (51.5)		77 (54.2)		159 (54.8)
Private[1]	236 (44.5)		56 (39.4)		117 (40.3)
Medicare-Medicaid		0.08		0.92	
No	318 (60)		73 (51.4)		146 (50.3)
Yes	212 (40)		69 (48.6)		144 (49.7)
Any Endocrinology Visit		0.02		0.12	
No	290 (54.7)		62 (43.7)		151 (52.1)
Yes	240 (45.3)		80 (56.3)		139 (47.9)
Number Medications - [Mean(SD)]	9.2(5.7)	<.001	11 (5.6)	0.06	10.1(5.7)
Number Diabetes Medications - [Mean(SD)]	0.8 (0.9)	0.62	0.8 (1)	0.60	0.8 (0.9)
Charlson Comorbidity Index [Mean(SD)]	5.7 (3.6)	<.001	7.7 (4)	0.32	7.1 (3.8)
Diabetes Severity Index [Mean(SD)]	4 (2.9)	<.001	5.5 (2.9)	0.28	5.1 (2.9)
Any Serious Mental Illness		0.01		0.25	
No	293 (55.3)		60 (42.3)		141 (48.6)
Yes	237 (44.7)		82 (57.7)		149 (51.4)
HbA1c [Mean(SD)]	6.9 (1.3)	0.01	7.6 (2.1)	0.60	7.24 (1.5)
SBP [Mean(SD)]	136.8 (14)	0.51	136.6(16.6)	0.30	137.6 (14.5)

1. All covariates are measured during the pre-index (baseline period). Propensity scores were generated using logistic regression models that included pre-index (HbA1c and SBP levels, age, gender, language preference, BMI category, smoking status, Charlson Co-morbidity index, Diabetes Severity Index, presence of serious mental illness (bipolar disorder, schizophrenia, major depression), having seen an endocrinologist ≥ 1 time, number of diabetes medications, total number of medications and health insurance status. Each UCMyRx-exposed patient was matched to two usual care patients using a Nearest Neighbor Matching approach. Bivariates were generated using t-test and Chi-squared test for continuous and categorical/dichotomous variables, respectively.

© 2023 Narain KC et al. *JAMA Network Open.*

eTable 7. Descriptive Statistics by Treatment Status for HbA1c Sample Exposed Hispanic vs Exposed Non-Hispanic White (Unmatched and Matched)					
**Covariates[1]**	**Source population for UCMyRx Whites (n= 168)**	***p*-value**	**UCMyRx Hispanics (n= 132)**	***p*-value**	**Matched Comparison n=132**
Age in years		0.03		0.79	
18-44	9 (5.4)		12 (9.1)		9 (6.8)
45-64	54 (32.1)		54 (40.9)		52 (39.4)
65-74	59 (35.1)		47 (35.6)		47 (35.6)
75+	46 (27.4)		19 (14.4)		24 (18.2)
Gender		0.11		0.46	
Female	84 (50)		79 (59.8)		72 (54.5)
Male	84 (50)		53 (40.2)		60 (45.5)
Smoking		1		1	
Non-Smoker	160 (95.2)		126 (95.5)		125 (94.7)
Currently Smoking	8 (4.8)		6 (4.5)		7 (5.3)
English Language		<.001		<.001	
No	10 (6)		68 (51.5)		10 (7.6)
Yes	158 (94)		64 (48.5)		122 (92.4)
BMI		0.46		0.69	
<18.5	1 (0.60)		0 (0)		0 (0)
18.5-24.9	31 (18.5)		17 (12.9)		21 (15.9)
25-29.9	47 (28)		41 (31.1)		36 (27.3)
>=30	89 (53)		74 (56.1)		75 (56.8)
Insurance		0.23		0.88	
Medicaid	15 (8.9)		11 (8.3)		13 (9.8)
Medicare	94 (56)		62 (47)		63 (47.7)
Private[1]	59 (35.1)		59 (44.7)		56 (42.4)
Medicare-Medicaid		<.001		<.001	
No	144 (85.7)		77 (58.3)		109 (82.6)
Yes	24 (14.3)		55 (41.7)		23 (17.4)
Any Endocrinology Visit		0.63		1	
No	43 (25.6)		38 (28.8)		37 (28)
Yes	125 (74.4)		94 (71.)		95 (72)
Number Medications - [Mean(SD)]	11.7 (6.2)	0.98	11.4 (6)	0.64	11.4 (6.3)
Number Diabetes Medications - [Mean(SD)]	1.1 (1)	0.34	1.2 (1.1)	0.96	1.2(1)
Charlson Comorbidity Index [Mean(SD)]	7.3 (3.8)	0.30	7.9 (4)	0.33	7.3 (4)
Diabetes Severity Index [Mean(SD)]	5.6 (3.2)	0.29	6 (3.3)	0.25	5.6 (3.4)
Any Serious Mental Illness		0.98		1	
No	65 (38.7)		50 (37.9)		50 (37.9)
Yes	103 (61.3)		82 (62.1)		82 (62.1)
HbA1c [Mean(SD)]	8.5 (1.9)	0.01	9.2 (2.2)	0.14	8.8 (2)
SBP [Mean(SD)]	133.3(16.1)	0.77	134(16.6)	0.89	133.6 (15.9)

1. All covariates are measured during the pre-index (baseline period). Propensity scores were generated using logistic regression models that included pre-index (HbA1c and SBP levels, age, gender, language preference, BMI category, smoking status, Charlson Co-morbidity index, Diabetes Severity Index, presence of serious mental illness (bipolar disorder, schizophrenia, major depression), having seen an endocrinologist ≥ 1 time, number of diabetes medications, total number of medications and health insurance status. Each UCMyRx-exposed patient was matched to two usual care patients using a Nearest Neighbor Matching approach. Bivariates were generated using t-test and Chi-squared test for continuous and categorical/dichotomous variables, respectively.

© 2023 Narain KC et al. *JAMA Network Open.*

eTable 8. Descriptive Statistics by Treatment Status for the Systolic Blood Pressure Sample Exposed Hispanic vs Exposed Non-Hispanic White (Unmatched and Matched)					
**Covariates[1]**	**Source population for UCMyRx Whites (n= 445)**	***p*-value**	**UCMyRx Hispanics (n= 265)**	***p*-value**	**Matched Comparison (n=265)**
Age in years		0.07		0.30	
18-44	18 (4)		18 (6.8)		18 (6.8)
45-64	135 (30.3)		96 (36.2)		117 (44.2)
65-74	146 (32.8)		83 (31.3)		73 (27.5)
75+	146 (32.8)		68 (25.7)		57 (21.5)
Gender		0.01		0.29	
Female	227 (51)		162 (61.1)		149 (56.2)
Male	218 (49)		103 (38.9)		116 (43.8)
Smoking		0.74		0.83	
Non-Smoker	430 (96.6)		254 (95.8)		252 (95.1)
Currently Smoking	15 (3.4)		11 (4.2)		13 (4.9)
English Language		<.001		<.001	
No	20 (4.5)		142 (53.6)		20 (7.5)
Yes	425 (95.5)		123 (46.4)		245 (92.5)
BMI		0.11		0.75	
<18.5	1 (0.2)		3 (1.1)		1 (0.4)
18.5-24.9	92 (20.7)		40 (15.1)		44 (16.6)
25-29.9	140 (31.5)		82 (30.9)		82 (30.9)
>=30	212 (47.6)		140 (52.8)		138 (52.1)
Insurance		0.14		0.09	
Medicaid	20 (4.5)		16 (6)		13 (4.9)
Medicare	248 (55.7)		128 (48.3)		106 (40)
Private[1]	177 (39.8)		121 (45.7)		146 (55.1)
Medicare-Medicaid		<.001		<.001	
No	388 (87.2)		167 (63)		213 (80.4)
Yes	57 (12.8)		98 (37)		52 (19.6)
Any Endocrinology Visit		0.36		0.30	
No	217 (48.8)		119 (44.9)		132 (49.8)
Yes	228 (51.2)		146 (55.1)		133 (50.2)
Number Medications - [Mean(SD)]	10 (6.2)	0.10	10.5 (5.6)	0.02	9.7(6.4)
Number Diabetes Medications - [Mean(SD)]	0.7 (0.9)	0.10	0.8 (1)	0.43	0.7 (0.9)
Charlson Comorbidity Index [Mean(SD)]	6.8 (3.942)	0.27	7.2 (3.9)	0.18	6.8 (4.2)
Diabetes Severity Index [Mean(SD)]	4.9 (3.049)	0.15	5.275 (3.2)	0.133	4.8 (3.2)
Any Serious Mental Illness		0.32		0.93	
No	170 (38.2)		112 (42.3)		114 (43)
Yes	275 (61.8)		153 (57.7)		151 (57)
HbA1c [Mean(SD)]	7.1 (1.7)	<.001	7.7 (2.1)	0.09	7.5 (2)
SBP [Mean(SD)]	133.8 (15.1)	0.23	136(16.8)	0.81	135.6 (14.3)

1. All covariates are measured during the pre-index (baseline period). Propensity scores were generated using logistic regression models that included pre-index (HbA1c and SBP levels, age, gender, language preference, BMI category, smoking status, Charlson Co-morbidity index, Diabetes Severity Index, presence of serious mental illness (bipolar disorder, schizophrenia, major depression), having seen an endocrinologist ≥ 1 time, number of diabetes medications, total number of medications and health insurance status. Each UCMyRx-exposed patient was matched to two usual care patients using a Nearest Neighbor Matching approach. Bivariates were generated using t-test and Chi-squared test for continuous and categorical/dichotomous variables, respectively.

© 2023 Narain KC et al. *JAMA Network Open.*
