# Supplementary material for: Pharmacist-Led Diabetes Control Intervention and Health Outcomes in Hispanic Patients With Diabetes
Source: JAMA Netw Open. 2023 Sep 28;6(9):e2335409. doi: 10.1001/jamanetworkopen.2023.35409 (PMC10539985; doi:10.1001/jamanetworkopen.2023.35409)
Supplement: Supplement 2. — Data Sharing Statement [file jamanetwopen-e2335409-s002.pdf]

Data Sharing Statement

Narain. Pharmacist-Led Diabetes Control Intervention and Health Outcomes in Hispanic Patients With Diabetes. *JAMA Netw Open*. Published September 25, 2023. doi:10.1001/jamanetworkopen.2023.35409

Data
Data available: No
